# Reinforced Feedback in Virtual Environment for Rehabilitation of Upper Extremity Dysfunction after Stroke: Preliminary Data from a Randomized Controlled Trial

**DOI:** 10.1155/2014/752128

**Published:** 2014-03-13

**Authors:** Paweł Kiper, Michela Agostini, Carlos Luque-Moreno, Paolo Tonin, Andrea Turolla

**Affiliations:** ^1^Laboratory of Kinematics and Robotics, IRCCS San Camillo Hospital Foundation, Via Alberoni 70, 30126 Venice, Italy; ^2^Physiotherapy Department, University of Seville, Calle San Fernando 4, 41004 Seville, Spain; ^3^Motion Analysis Laboratory, “Virgen del Rocio” Hospital, Physiotherapy Area, Avenida Manuel Siurot, 41013 Seville, Spain

## Abstract

*Objectives*. To study whether the reinforced feedback in virtual environment (RFVE) is more effective than traditional rehabilitation (TR) for the treatment of upper limb motor function after stroke, regardless of stroke etiology (i.e., ischemic, hemorrhagic). *Design*. Randomized controlled trial. *Participants*. Forty-four patients affected by stroke. *Intervention*. The patients were randomized into two groups: RFVE (*N* = 23) and TR (*N* = 21), and stratified according to stroke etiology. The RFVE treatment consisted of multidirectional exercises providing augmented feedback provided by virtual reality, while in the TR treatment the same exercises were provided without augmented feedbacks. *Outcome Measures*. Fugl-Meyer upper extremity scale (F-M UE), Functional Independence Measure scale (FIM), and kinematics parameters (speed, time, and peak). *Results*. The F-M UE (*P* = 0.030), FIM (*P* = 0.021), time (*P* = 0.008), and peak (*P* = 0.018), were significantly higher in the RFVE group after treatment, but not speed (*P* = 0.140). The patients affected by hemorrhagic stroke significantly improved FIM (*P* = 0.031), time (*P* = 0.011), and peak (*P* = 0.020) after treatment, whereas the patients affected by ischemic stroke improved significantly only speed (*P* = 0.005) when treated by RFVE. *Conclusion*. These results indicated that some poststroke patients may benefit from RFVE program for the recovery of upper limb motor function. This trial is registered with NCT01955291.

## 1. Introduction

Stroke is one of the most serious neurological disorders rated as the third cause of death worldwide [1]. Epidemiological data indicates a mortality of 30% in the first month after stroke independently from the type of cerebrovascular injury, while only 10% of patients was discharged from hospital without serious functional or cognitive impairments [2]. Among the survivors to a first stroke onset, 73% to 88% result in acute hemiparesis [3]. Indeed, the disruption of motor function is a major source of impairment affecting both upper and lower limbs, frequently impeding autonomy in the activities of daily living (ADL) [4, 5]. It was estimated that at least 60% of the patients affected by stroke present severe reduction in the ability to perform ADL [4, 6, 7], requiring an intensive rehabilitation care particularly focused on the recovery of the upper limb motor function.

Several studies using fMRI and transcranial magnetic stimulation (TMS) in humans provided evidence that functional adaptation of the motor cortex following stroke is still possible [4, 8–12]. Changes of cerebral activation in the sensory and motor systems occur early after stroke and may be a first step toward the restoration of motor function. Furthermore, many studies have demonstrated that neuroplasticity can occur even in case of chronic stroke [4, 13]. In fact, it was noted that task-oriented exercises induce regenerative capacities of the central nervous system (CNS), in poststroke patients [8, 14]. It was also noted that plasticity of the CNS, thus its adaptability to natural developmental changes [14, 15], is maintained lifelong regardless of age [16].

The traditional rehabilitation approaches based on one-to-one physiotherapist-patient interaction are the most widespread for the treatment of the upper extremity in clinical settings and its effectiveness was demonstrated by several studies [17–19]. However, recent evidence is enlightening the possibility that innovative approaches, based on the augmentation of specific kinematic feedbacks, could enrich the rehabilitation environment, possibly leading to a significant improvement of the motor function [20–24]. Innovative technologies have provided the opportunity to enrich the environments where motor rehabilitation program is carried out. This enrichment could potentially facilitate the physiological activation of the brain areas devoted to motor relearning. Following these principles, exercises should involve multiple sensory modalities exploiting the adaptive nature of the nervous system, in order to promote active patient participation [25]. Previous evidence has demonstrated that training in virtual environment promotes learning in normal subjects, as well as in poststroke patients, underpinned by the providing of augmented feedbacks related to motor performance and result [26–30]. Moreover, other effects dependent on the interaction with virtual environments were measured at cortical activation level using functional magnetic resonance imaging (fMRI). To date, neuroimaging evidence showed that reorganization of the motor cortex and related motor recovery were changed significantly after virtual reality based treatments [9, 13, 31]. As a consequence, reinforced feedback in virtual environment (RFVE) can promote the recovery of motor function in poststroke patients, by means of regular, intensive, and supervised training [5, 32, 33].

The first aim of the present study was to determine whether the RFVE was more effective than traditional rehabilitation (TR) treatments for the recovery of upper limb motor function after stroke. The second aim was to study whether any difference exists on the effect of RFVE, due to stroke etiology (i.e., hemorrhagic, ischemic).

## 2. Methods

### 2.1. Study Design and Participants

A single blind randomized trial was run considering as eligible the inpatients accepted at the Neurorehabilitation Department of IRCCS San Camillo Hospital Foundation (Venice, Italy). The patients enrolled were randomized in two groups (RFVE or TR) according to a simple randomization technique. In the RFVE group the patients were treated one hour a day by the experimental treatment and one hour a day by TR treatment. In the TR group the patients underwent two hours daily of TR training. Both treatments lasted 5 days weekly for 4 weeks.

The inclusion criterion for patients enrollment was the diagnosis of a first stroke (ischemic or hemorrhagic) occurring at last 1 year before the enrollment and never treated before with RFVE. The exclusion criteria were clinical evidence of severe cognitive impairment (i.e., a score lower than 24 points at the Mini-Mental State Examination), clinical history of neglect, the presence of complete hemiplegia (i.e., Fugl-Meyer upper extremity scale = 0 pts.), sensory disorders (i.e., a score lower than 16 points at sensibility subitem of the Fugl-Meyer scale), and history of traumatic injuries (e.g., fracture, joint dislocation with permanent dysmorphism after trauma) impairing the upper limb motor function.

The institutional review board of the IRCCS San Camillo Hospital Foundation approved the study protocol. All patients were informed about the aim and procedures of the study and written informed consent was obtained from all participants.

### 2.2. Interventions

**Figure 1:**
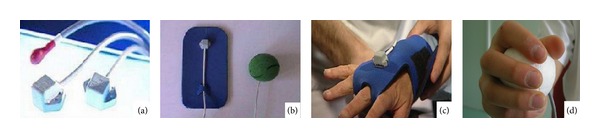
(a) Type of receivers, (b) application of receivers to different end-effectors, (c) sensorised glove for the application of receiver in case of severe motor deficit, and (d) modality of end-effector application in case of grasping being preserved.

**Figure 2:**
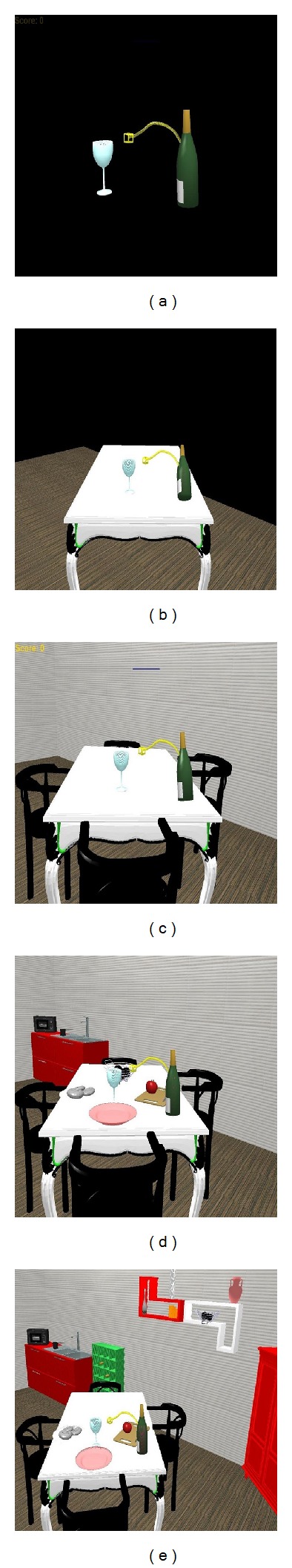
The same motor task represented from lowest to highest complexity.

The experimental and control rehabilitation programs lasted two hours a day, for five days a week, for four weeks. The patients allocated to RFVE group, were treated using the “Virtual Reality Rehabilitation System” (VRRS-Khymeia Group, Ltd., Noventa Padovana, Italy) composed by a PC workstation connected to a 3D motion-tracking system (Pohlemus LIBERTY Colchester, Vermont, US) and a high-resolution LCD projector displaying the virtual scenarios on a large wall screen. During the virtual reality treatment the subject was seated in front of the wall screen grasping a sensorized real object (i.e., ball, disc, or glass) with the paretic hand; in case of severe impairment of grasping the sensor was fixed to a glove worn by the patient (Figure 1). The real object, held by the subject, was matched to the virtual object displayed on the wall screen through an electromagnetic sensor placed onto the dorsal face of the hand (i.e., end-effector). The virtual scenarios could be created by the physiotherapist recording the movements carried out by himself while grasping the same sensorized object used for the patients. In the virtual scenario, the therapist determined the location of the starting position, the target to reach for each task, and the path to follow. Additionally, virtual obstacles in the arm workspace could be displayed with the aim of increasing the complexity of the motor task. A simple reaching movement could accomplish just a straight path, whereas others required more complex movements (Figure 2). Hence, the therapist created a sequence of motor tasks that the patient was asked to perform on his workstation along the therapy session. The physiotherapist determined the complexity of the task, tailored on patient's motor deficit.

The patients allocated in the TR group, were treated with the aim of reducing impairments and improving ADLs. Traditional stroke rehabilitation programs emphasize functional training to promote the individual recovery and to maintain the subjects as much independent as possible. The traditional rehabilitation for the upper extremity consisted of exercises of various movements in a horizontal or vertical plane. Also in the case of TR treatment the rehabilitation program was planned in accordance with the patients' current capacity. For each patient individual exercises were selected with progressive complexity and they were asked to perform exercises for postural control, exercises for hand preconfiguration, exercises for the stimulation of manipulation and functional skills, and exercises for proximal-distal coordination. All the exercises were performed with or without the assistance of a physiotherapist. To achieve the requested goal patients were asked to perform various movements, such as shoulder flexion and extension, shoulder abduction and adduction, shoulder internal and external rotation and shoulder circumduction, elbow flexion and extension, forearm pronation and supination, and hand grasping-release and clenching into a fist. We introduced exercises such as, for example, the following: to strengthen the shoulder abductors patient was asked to abduct their shoulder with their elbow extended, to strengthen the shoulder flexors the patient started with their arm down beside their body and finished the movement with their arm above the head, (movement was performed keeping the elbow straight), to improve the ability to reach the objects the patient was instructed to pick up the object and place it on the table in front of them and then put it back again, and to stretch or maintain range of the wrist joint the subject was asked to supinate and pronate their wrist through full range of motion according to the requested task.

### 2.3. Outcome Measures

The functional assessment included the Fugl-Meyer upper extremity (F-M UE) scale and the Functional Independence Measure (FIM) as measurements of motor function and independence, respectively. Furthermore, we conducted a kinematics analysis of the paretic arm considering as outcomes the mean linear velocity (speed), the mean duration of movements (time), and the mean number of submovements (peak). The kinematic assessment consisted of eight exercises (i.e., elbow extension, elbow flexion, shoulder adduction, reaching movement, forearm pronation/supination, shoulder abduction, shoulder flexion, and shoulder internal/external rotation), each one repeated 10 times. Patients were informed about the movement aim and sample movements were performed before the measurement to familiarize with the system. All the functional and kinematics assessments were conducted at the beginning and at the end of the treatment in both groups. The requested tasks during kinematic assessment were different from those used for RFVE treatment and TR training.

### 2.4. Data Analysis

The distribution skewness was studied with the Kolmogorov-Smirnov test and according to the results parametric or nonparametric tests were used to determine if the outcomes were statistically different in the comparison within and between groups. The enrolled patients were stratified a posteriori according to stroke etiology (i.e., ischemic, hemorrhagic) both in experimental and control groups. A subgroup analysis was run on the strata resulted for comparing any significant difference in the considered outcomes due to the kind of stroke. Statistical significance was set at *P* < 0.05 and IBM SPSS 20.0 package software was used for the analysis.

## 3. Results

**Figure 3:**
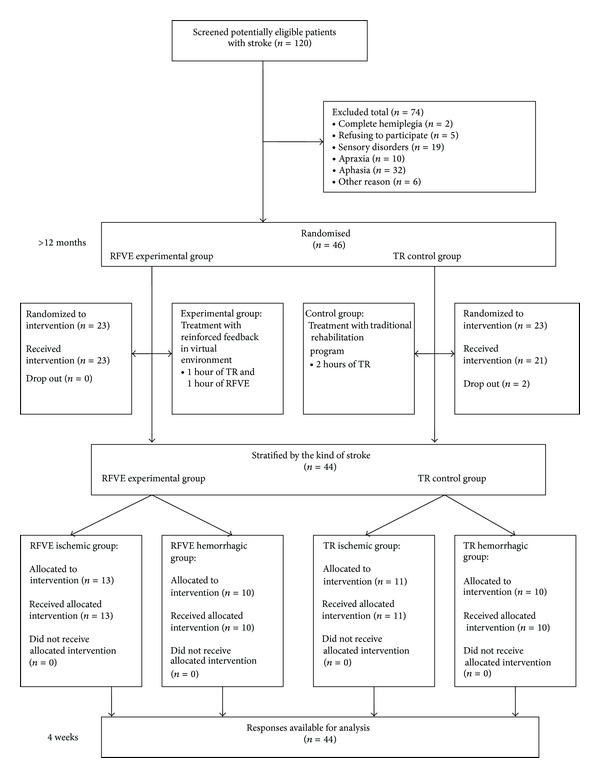
Flowchart of participants through the study.

A group of 120 eligible patients accomplishing the inclusion/exclusion criteria was screened; among them 46 were enrolled for randomization and allocated to RFVE (*n* = 23) and TR (*n* = 23) groups, respectively. During the study 2 patients dropped out from the TR group because they were discharged from the hospital earlier. Thus, data from 44 subjects that completed the intervention were included for the analysis. The complete flow of the trial is reported in Figure 3.

The overall group consisted of 29 (66%) men and 15 (34%) women, 24 (55%) patients affected by ischemic stroke and 20 (45%) by hemorrhagic stroke. The participants had a mean age of 64.3 ± 12.6 years and were enrolled in the study at a mean distance from stroke of 4.2 ± 3.1 months. All patients reported to be comfortable throughout the training and did not experience any side effect caused by interaction with virtual environment (e.g., nausea, dizziness, headache, disorientation) [34].

The RFVE group consisted of 13 (57%) ischemic and 10 (43%) hemorrhagic stroke patients, 14 (61%) men and 9 (39%) women. The mean age was 63.1 ± 9.5 while the mean distance from stroke onset was 3.7 ± 2.3 months. The TR group consisted of 15 (71%) men and 6 (29%) women; moreover, 11 (52%) were affected by ischemic stroke and 10 (48%) by hemorrhagic stroke. The patients' mean age was 65.5 ± 14.2 years, and mean time since stroke onset was 4.8 ± 3.6 months.

All the outcomes were comparable between groups (i.e., F-M UE, *P* = 0.306; FIM, *P* = 0.329; time, *P* = 0.768; speed, *P* = 0.590; peak, *P* = 0.841), at baseline. Also the demographics characteristics were comparable (i.e., time from stroke, *P* = 0.206; age, *P* = 0.289; sex, *P* = 0.602).

Considering the overall groups, the results showed that FIM changed significantly after both treatments (RFVE group: *P* = 0.001; TR group: *P* = 0.006), while F-M UE scale improved significantly after RFVE training (*P* = 0.001) but not after TR treatment (*P* = 0.053). All the kinematics outcomes changed significantly after RFVE (time, *P* = 0.001; speed, *P* = 0.001; peak, *P* = 0.001) and TR (time, *P* = 0.028; speed, *P* = 0.018; peak, *P* = 0.045) treatments (Table 1).

The RFVE treatment showed to be significantly more effective than TR treatment, as measured by F-M UE (RFVE: 10.3%, TR: 4.8%; *P* = 0.030), FIM (RFVE: 12.5%, TR: 6.4%; *P* = 0.021), time (RFVE: 41.0%, TR: 15.6%; *P* = 0.008), and peak (RFVE: 26.1%, TR: 21.1%; *P* = 0.018), while speed did not change significantly between groups after therapy (RFVE: 35.7%, TR: 20.5%; *P* = 0.140).

With regard to subgroup analysis of patients following hemorrhagic stroke, all the outcomes improved significantly after the RFVE treatment (i.e., F-M UE, *P* = 0.012; FIM, *P* = 0.005; time, *P* = 0.001; speed, *P* = 0.001; peak, *P* = 0.001), while only FIM (*P* = 0.035), time (*P* = 0.001), and speed (*P* = 0.001) changed significantly after TR treatment (Table 1). Moreover, RFVE gained better results than TR treatment at FIM (RFVE: 17.5%, TR: 10.2%; *P* = 0.031) and time (RFVE: 41.5%, TR: 23.5%; *P* = 0.034).

With regard to subgroup of patients affected by ischemic stroke all the outcomes improved significantly after RFVE treatment (F-M UE, *P* = 0.009; FIM, *P* = 0.004; time, *P* = 0.001; speed, *P* = 0.001; peak, *P* = 0.001), while only speed (*P* = 0.030) and peak (*P* = 0.004) changed significantly after the TR treatment (Table 1). Moreover, only speed was significantly better after RFVE (40.5%) than TR (17.3%) treatments (*P* = 0.005). Finally, only FIM scale (*P* = 0.035) was significantly different between ischemic and hemorrhagic stroke patients undergoing RFVE treatment.

## 4. Discussion

In this study, we compared the effects of an innovative rehabilitation modality called reinforced feedback in virtual environment with the ones gained by traditional rehabilitation treatment for the recovery of upper limb motor function after stroke. The results demonstrated the therapeutic effect of the RFVE treatment, sustaining the beneficial integration with the TR treatment. When combined, the TR and the RFVE treatments seem to regain a better motor function compared to the recovery induced by the simple augmenting of the conventional rehabilitation program intensity. The RFVE showed significant better effects than conventional treatment, confirming the evidence coming from previous studies [27, 29, 32, 35]. Indeed, also kinematic outcomes showed a significant improvement, after the RFVE than the TR treatment. Moreover at the end of both treatments patients were asked to complete the satisfaction questionnaire. The result provided positive patients' feedback regarding the performance of 2 hours of treatment in both groups. The absence of statistically significant improvement within TR group in F-M UE scale may be explained as a result of patients dropped out from the control group. Moreover, the results from subgroup analysis showed contradictory results compared with findings from overall groups. The time from stroke was comparable between groups, but it should be acknowledged that a mean difference over 1 month may be clinically relevant, requiring a stratified analysis according to distance since stroke onset for future reporting of the complete trial. Considering these limitations, patients following hemorrhagic stroke seem to take greater advantage from RFVE treatment than ischemic and TR patients. In addition, data demonstrated that the effect of RFVE therapy is beneficial independently from stroke etiology (i.e., hemorrhagic, ischemic) for both motor and ADL functions.

Although the neural mechanisms associated with practice dependent motor recovery are not clearly understood, it has been suggested that intensive and repetitive use of the affected limb, as those stimulated by RFVE, may induce positive effect on neuroplasticity and improvement of motor function [5, 36]. Movement relearning implies a process of motor actions' selection in order to perform the requested task. We argued that in our proposal different paradigms (e.g., reinforcement learning, supervised learning) operate to promote motor learning, based on the feedbacks received from the artificial environment. In reinforcement learning the subject estimates directly meaningful information on the performed movement. This learning is based on knowledge of the results (KR), which in the RFVE training is represented by the score of each single movement, and on knowledge of the performance (KP), which in the RFVE training is represented by the trajectory of every trial displayed at the end of every session. In supervised learning paradigm the subject receives from a teacher a prompt to adjust the movement execution; in RFVE training such feedback is represented by a “virtual teacher” showing the correct movement execution in real time [37–39]. Based on these paradigms, we hypothesized that the treatment in virtual environment has an impact on the functioning of the upper limb. The motor and functional changes observed may have led to a reorganization of the cerebral cortex related to the affected limb.

The results from this study, even if preliminary, demonstrated the potential impact of the use of technology in clinical settings to tailor the rehabilitation sessions with the aim of increasing the intensity and specificity of practice. The findings demonstrated the feasibility of using RFVE to improve meaningfully the outcomes along the rehabilitation process after stroke. According to our sample size calculation (with F-M UE scale as primary outcome) a minimum of 136 subjects should be enrolled to complete the study (*α* = 0.05 and *β* = 0.8).Therefore, a bigger sample and more complex RFVE tasks are needed to confirm our preliminary data and to validate the findings.

## 5. Conclusion

Our results revealed that the application of augmented feedback by means of RFVE treatment combined with the TR program is more effective than the same amount of conventional rehabilitation treatment to reduce the upper limb dysfunction after stroke. The RFVE treatment augments the effects of the upper limb movement supporting the reacquisition of accurate motor control. The positive results indicate that the application of RFVE treatment is promising to reduce the impairment of the upper limb and may be clinically relevant for stroke rehabilitation.

## Figures and Tables

**Table 1:** Effects of experimental and control treatments on functional and kinematics outcomes.

	RFVE		TR	
	Pre-test	Post-test	Pre-test	Post-test
*Overall *				
Functional				
F-M UE	43.0 ± 14.7 (36.64–49.36)	49.8 ± 12.5^∗§^ (44.42–55.32)	46.3 ± 17.5 (38.36–54.31)	49.5 ± 16.2 (42.13–56.92)
FIM	87.6 ± 29.6 (74.82–100.48)	103.3 ± 22.9^∗§^ (93.44–113.26)	96.6 ± 24.6 (85.35–107.80)	104.6 ± 18.2* (96.32–112.91)
Kinematics				
Time (s)	11.7 ± 17.4 (9.75–13.61)	6.9 ± 8.4^∗§^ (5.94–7.80)	9.6 ± 13.2 (8.03–11.27)	8.1 ± 11.1* (6.73–9.45)
Speed (cm/s)	15.7 ± 6.9 (14.93–16.46)	21.3 ± 11.2^∗§^ (CI 20.04–22.52)	15.6 ± 7.1 (14.74–16.48)	18.8 ± 10.6* (17.54–20.14)
Peak (*n*)	13.4 ± 12.6 (12.03–14.81)	9.9 ± 12.6* (8.53–11.32)	12.8 ± 11.6 (11.39–14.22)	10.1 ± 12.8* (9.36–12.50)
*Posthemorrhagic group outcomes *				
Functional				
F-M UE	38.6 ± 19.3 (24.74–52.46)	47.6 ± 15.1* (36.79–58.41)	47.6 ± 17.5 (35.03–60.17)	51.5 ± 16.4 (39.75–63.25)
FIM	71.0 ± 32.2 (47.96–94.04)	93.1 ± 28.8^∗§^ (72.49–113.71)	94.8 ± 26.0 (76.18–113.42)	107.7 ± 17.2* (95.37–120.03)
Kinematics				
Time (s)	13.5 ± 18.9 (10.40–16.69)	7.9 ± 9.0^∗§^ (6.44–9.42)	8.5 ± 7.0 (7.21–9.82)	6.5 ± 7.2* (5.18–7.86)
Speed (cm/s)	15.1 ± 6.7 (14.40–16.63)	20.1 ± 10.1* (18.44–21.81)	16.3 ± 7.1 (15.02–17.65)	20.5 ± 9.3* (18.73–22.18)
Peak (*n*)	15.4 ± 13.7 (13.17–17.73)	11.6 ± 12.7* (9.54–13.77)	13.0 ± 12.6 (10.67–15.34)	11.2 ± 11.9 (8.99–13.40)
*Postischemic group outcomes *				
Functional				
F-M UE	46.3 ± 9.2 (40.77–52.0)	51.6 ± 10.5* (45.22–58.01)	45.1 ± 18.2 (32.92–57.44)	47.7 ± 16.6 (36.53–58.92)
FIM	100.4 ± 20.6 (87.98–112.95)	111.2 ± 13.6* (103.0–119.46)	98.1 ± 24.4 (81.73–114.64)	101.8 ± 19.4 (88.75–114.89)
Kinematics				
Time (s)	10.1 ± 16.0 (7.75–12.55)	6.0 ± 7.8* (4.83–7.17)	10.5 ± 16.5 (7.83–13.26)	9.3 ± 13.3 (7.13–11.50)
Speed (cm/s)	15.8 ± 7.1 (14.79–16.91)	22.2 ± 12.0^∗§^ (20.43–24.02)	15.0 ± 7.0 (13.88–16.20)	17.6 ± 11.4* (15.70–19.43)
Peak (*n*)	11.7 ± 11.3 (10.06–13.45)	8.5 ± 12.3* (6.65–10.35)	12.6 ± 10.7 (10.88–14.40)	10.7 ± 13.5* (8.50–12.95)

Data are displayed as mean and standard deviation and 95% confidence interval in brackets.

RFVE: reinforced feedback in virtual environment training; TR: traditional rehabilitation treatment; F-M UE: Fugl-Meyer upper extremity; FIM: functional independence measure; time: mean duration of movement; speed: mean velocity of movement; peak: mean number of submovement; *P < 0.05 within group analysis (Wilcoxon test); ^§^ *P* < 0.05 between group analysis (Mann–Whitney *U* test).
